# Expression profiling of colorectal cancer cells reveals inhibition of DNA replication licensing by extracellular calcium^☆^

**DOI:** 10.1016/j.bbamcr.2017.01.017

**Published:** 2017-06

**Authors:** Abhishek Aggarwal, Herbert Schulz, Teresa Manhardt, Martin Bilban, Rajesh V Thakker, Enikö Kallay

**Affiliations:** a Department of Pathophysiology and Allergy Research, Medical University of Vienna, Austria; b Cologne Center for Genomics (CCG), Cologne, Germany; c Department of Medical and Chemical Laboratory Diagnostics, Medical University Vienna, Austria; d Radcliffe Department of Medicine, University of Oxford, UK

**Keywords:** Calcium, Colorectal cancer, DNA replication licensing, Minichromosome maintenance complex, Calcium-sensing receptor

## Abstract

Colorectal cancer is one of the most common cancers in industrialised societies. Epidemiological studies, animal experiments, and randomized clinical trials have shown that dietary factors can influence all stages of colorectal carcinogenesis, from initiation through promotion to progression. Calcium is one of the factors with a chemoprophylactic effect in colorectal cancer. The aim of this study was to understand the molecular mechanisms of the anti-tumorigenic effects of extracellular calcium ([Ca^2+^]_o_) in colon cancer cells.

Gene expression microarray analysis of colon cancer cells treated for 1, 4, and 24 h with 2 mM [Ca^2+^]_o_ identified significant changes in expression of 1571 probe sets (ANOVA, *p* < 10^− 5^). The main biological processes affected by [Ca^2+^]_o_ were DNA replication, cell division, and regulation of transcription. All factors involved in DNA replication-licensing were significantly downregulated by [Ca^2+^]_o_. Furthermore, we show that the calcium-sensing receptor (CaSR), a G protein-coupled receptor is a mediator involved in this process. To test whether these results were physiologically relevant, we fed mice with a standard diet containing low (0.04%), intermediate (0.1%), or high (0.9%) levels of dietary calcium. The main molecules regulating replication licensing were inhibited also *in vivo*, in the colon of mice fed high calcium diet.

We show that among the mechanisms behind the chemopreventive effect of [Ca^2+^]_o_ is inhibition of replication licensing, a process often deregulated in neoplastic transformation. Our data suggest that dietary calcium is effective in preventing replicative stress, one of the main drivers of cancer and this process is mediated by the calcium-sensing receptor.

## Highlights

• Extracellular calcium inhibits cell division, DNA replication, in colon cancer cells.
• Extracellular calcium inhibits replication licensing *in vitro* and *in vivo*.
• The calcium-sensing receptor is a critical mediator of this process.

## 1 Introduction

Due to ageing of the population and increased life expectancy, cancer in the elderly has become an increasing problem in the Western world. In Europe, colorectal cancer (CRC) is the second most common cause of cancer morbidity and mortality [1]. According to GLOBOCAN, 1,360,602 new cases of, and 693,933 deaths from CRC were estimated worldwide in 2012 [2]. The impact of diet on both the initiation and progression of cancer becomes increasingly evident. The World Health Organisation considers that after smoking, dietary habits constitute the second most preventable cause of cancer. Several prospective cohort studies [3], [4], as well as large case-control studies and randomized trials showed that higher intake of dietary calcium is associated with lower risk of CRC [5], [6], [7], [8], [9], [10], [11] identifying nutritional calcium as a promising chemopreventive agent. The mechanisms underlying the chemopreventive effects of calcium are partly mediated by binding and eliminating potentially mitogenic bile acids and free fatty acids [12], [13], [14]. However, calcium is able to inhibit proliferation [15], [16], [17] and induce both, differentiation and apoptosis in colonocytes directly [18], [19], [20] without the involvement of fat.

There is evidence that calcium supplementation prevents both development [21] and recurrence of colon adenomas (*e.g.* [22]) and it reduces rectal epithelial cell proliferation in patients at high risk for developing cancer of the colon [15]. The NIH-AARP Diet and Health Study reported that dairy food and high calcium consumption was inversely associated with cancers of the digestive system, and that decreased risk was particularly pronounced in colorectal cancer patients [10]. Studies of a preclinical model of sporadic colorectal cancer have reported that a Western-style diet (high in dietary fat, low in calcium and vitamin D) induced formation of colonic tumors, but that supplementation with calcium and vitamin D resulted in significant decrease in both tumor incidence and multiplicity [23], [24], [25]. We have shown previously that dietary calcium reduced proliferation of mouse colonic crypts [16], [17], [26].

In order to exploit calcium as a chemopreventive agent it is of utmost importance to understand the molecular mechanisms by which dietary calcium reduces risk of colorectal cancer and find markers that would determine whether a person would respond to calcium treatment [19].

A well-accepted model to study intestinal epithelial cell proliferation, differentiation, and apoptosis is the human colon adenocarcinoma-derived Caco-2 cell line [27]. These cells are able to differentiate spontaneously in culture. This differentiation mimics, at least partially, the phenotypic changes along the absorptive cell lineage of the intestine [28], [29], [30]. Our previous research showed that extracellular calcium [Ca^2+^]_o_ is a powerful inhibitor of proliferation in these cells [31] and that this effect is mediated, at least in part, by the calcium-sensing receptor (CaSR), a G-protein coupled receptor [32]. In order to determine the molecular pathways that are affected by [Ca^2+^]_o_, we evaluated gene expression in Caco-2 cells after treatment with 2 mM [Ca^2+^]_o_ by microarray analysis. To determine whether these results were physiologically relevant, we tested whether high (0.9%) calcium diet would have similar effects in the colon of mice. Our studies revealed, that calcium regulates critical steps of the replication licensing both *in vitro* and *in vivo* and that the CaSR is a critical mediator involved in this process.

## 2 Materials and methods

### 2.1 Cell culture

We have used two colon cancer cell lines: the Caco-2/AQ cells, which are growth-inhibited by [Ca^2+^]_o_ and express the CaSR [32], and the HT-29 cells, which are unresponsive to the antiproliferative effect of [Ca^2+^]_o_ treatment [33] and express undetectable levels of CaSR [34].

Caco-2/AQ cells, a subclone of the colorectal adenocarcinoma cell line Caco-2, were cultured as previously described [34], [35]. Cell line authentication was performed using STR profiling (DNA Diagnostics Centre, UK) and cells were routinely screened for mycoplasma contamination using the VenorGem Mycoplasma Detection Kit (Minerva Biolabs, Germany).

Two days after confluence the cells were exposed to serum-free, calcium-free DMEM supplemented with 5 mg/ml insulin, 5 mg/ml transferrin, 5 ng/ml sodium selenite (ITS, LifeTechnologies, UK) as previously described [16] for 48 h after which the cells were treated with 2 mM [Ca^2+^]_o_ for 1, 4, and 24 h.

To study the role of the CaSR, we used the HT29 colon cancer cells stably expressing the full length CaSR (HT29^CaSR^) or an empty vector (HT29^EMP^) as previously described [34], [36].

### 2.2 RNA extraction and sample preparation for microarray analysis

Total RNA was extracted with TRIzol® reagent (Invitrogen Ltd. Paisley, UK) according to the manufacturer's instructions, purified with QIAGEN's RNeasy Total RNA Isolation kit (Qiagen GmbH, Germany) and quantified using Nanodrop ND-1000. The quality of the RNA was assessed by agarose-formaldehyde gel electrophoresis. We synthesized double-stranded cDNA from 5 μg total RNA with the cDNA synthesis system kit (Roche, Switzerland).

### 2.3 Microarray and data analysis

**Supplementary Fig. 1:**
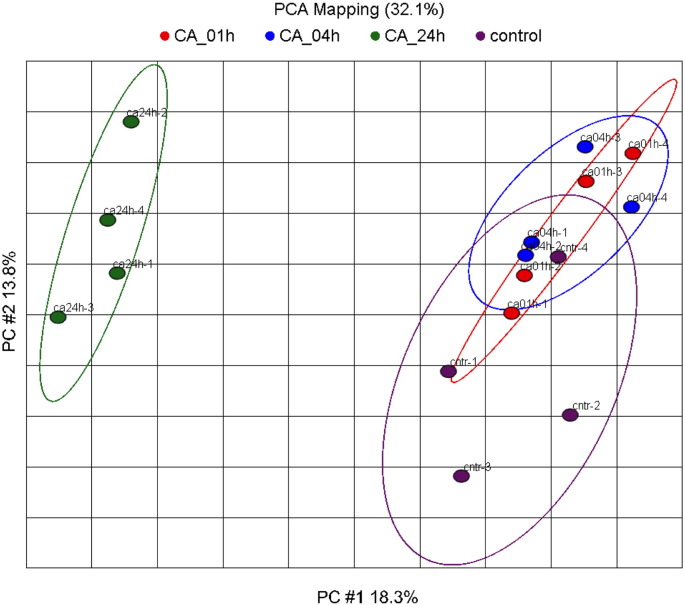
Result of the PCA mapping of all transcripts. Principal component one (X-axis) and two (Y-axis) are plotted. These two PCs describe 32.1% of the dataset variance. In PC #1 mainly the 24 h CA treatment condition is separated from all other conditions.

Biotinylated cRNA was synthesized with Perkin-Elmer's nucleotide analogs using the Ambion's MEGAScript T7 kit. For target preparation cRNA was fragmented with the standard Affymetrix protocol. Fragmented cRNA was hybridized for 16 h at 45 °C to the Human genome 133 plus 2 Array (Affymetrix, UK), which includes 54,675 probe-sets. Following hybridization, arrays were washed and stained with streptavidin-phycoerythrin in the Affymetrix Fluidics Station 400 and further scanned using the Hewlett-Packard GeneArray Scanner G2500A. Image data were analyzed with GCOS 1.4 using default analysis settings from Affymetrix and global scaling as normalization method. All chips passed the quality criteria. Comparability of the experiment conditions among the treatment groups was checked by Principal component analysis using Partek Genomic Suite (6.3 beta) correlation as a dispersion matrix and normalized Eigenvector scaling (Suppl. Fig. 1).

After robust multi-array average (RMA) normalization [37], Analysis of Variance (ANOVA) was performed. The false discovery rate of each test-set was calculated using the Benjamini Hochberg procedure [38].

We have analyzed the differentially expressed probe-sets with DAVID (Database for Annotation, Visualization and Integrated Discovery) and with the Ingenuity Pathway Analysis (IPA) tool. DAVID is a free online bioinformatics resource that provides a comprehensive set of functional annotation tools to understand biological meaning behind large list of genes by condensing large gene lists into gene functional groups [39], [40], [41]. IPA is a web-based software application that enables analysis, integration, and understanding of data from gene expression experiments. We used the IPA tool to assign the differentially expressed probe-sets to common biological pathways and biological functions. The right-tailed Fisher's exact test was used to measure the significance of the association between each gene list and a canonical pathway.

### 2.4 Quantitative reverse-transcriptase Polymerase Chain Reaction (qRT-PCR)

Expression analysis of target genes was performed by quantitative reverse-transcriptase Polymerase Chain Reaction (qRT-PCR). Total RNA was extracted using TRIzol® reagent (LifeTechnologies) and cDNA was reverse-transcribed as previously described [42]. qRT-PCR was performed using Power SYBR® Green PCR Master Mix on a StepOne Plus qRT-PCR device (LifeTechnologies). Where possible, primers were designed to span different exons to prevent amplification of potentially contaminating genomic DNA. Relative expression (ΔΔC_t_) of target genes was normalized to endogenous control genes; large ribosomal protein (hRPLPO) for the human cell lines, and eukaryotic translation elongation factor 1 beta 2 (mEef1b2) and beta Actin (mβ-Actin) for the mouse tissue [43], and calculated relative to untreated control cells or a commercially available total RNA calibrator (Clontech, USA). At least three different experiments were set up for each sample and transcript under investigation. All primers used in this study have been previously described [34].

### 2.5 Western blot

Protein isolation and western blotting were performed as described previously [44]. Antibodies used were against: CDT1 (kind gift from Dr. Zoi Lygerou, University of Patras, Greece), CDC6 and CDC45 (Santa Cruz Biotechnology, USA), MCM2 (Abcam, UK), and Actin (Sigma-Aldrich, USA).

### 2.6 Immunofluorescence

Treated cells were fixed with 4% paraformaldehyde for 30 min and permeabilized with 0.2% TritonX-100 in PBS. Unspecific fluorescence was blocked with 3% BSA/0.1% TritonX-100 in PBS for 30 min. Primary antibody diluted in blocking solution was applied over night at 4 °C. After washing with 0.2% BSA in PBS, cells were incubated with the secondary antibodies; either goat anti-rabbit Alexa-Fluor 488- or goat anti-mouse Alexa Fluor 568-labeled IgG for 1 h at room temperature. Nuclei were stained with DAPI. Isotype-matched IgG was used as negative control.

### 2.7 Animals used in the dietary calcium experiment

C57BL/6 mice (at least 10/group) were housed in the Center for Laboratory Animal Care of the Medical University of Vienna in a contained environment. Study protocol was approved by the Committee of Animal Experimentation of the Medical University of Vienna and by the Austrian Ministry of Science and Education. Mice were fed a standard rodent diet (AIN 76A diet) containing 0.9%, 0.1% or 0.04% calcium. Whole colons were cut open, rinsed with ice-cold PBS, and snap frozen in liquid nitrogen and stored at − 80 °C until analysis.

### 2.8 Statistical analysis

Statistical differences between two groups were analyzed with Student's *t*-test. Analysis of Variance (ANOVA) test followed by Tukey's post-test was used for group comparisons. *p* values < 0.05 were considered statistically significant. GraphPad prism (GraphPad Software Inc., USA) was used for all statistical calculations and for plotting graphs.

## 3 Results

### 3.1 Identification of differentially expressed transcripts

A gene expression array analysis of 54,675 probe-sets was conducted to compare changes between cells treated with 2 mM [Ca^2+^]_o_ for 1 h, 4 h and 24 h (Suppl. Table 1). Principal component analysis (PCA) (based on 2 × standard deviation of the controls) revealed that the groups treated with 2 mM [Ca^2+^]_o_ for 1 h (ca01h) or 4 h (ca04h) were not totally separated from the controls suggesting no significant effect of the calcium treatment. Significant differences were detected mainly after 24 h of treatment (Suppl. Fig. 1).

The time dependent effect of [Ca^2+^]_o_ on gene expression was analyzed by ANOVA. Applying highly stringent conditions, only changes with a false discovery rate (FDR) value < 10^−3^ in the ANOVA calculation were considered to be significantly influenced by [Ca^2+^]_o_ treatment. These 1571 differentially expressed probe-sets were selected for hierarchical clustering and further analysis.

**Supplementary Fig. 2:**
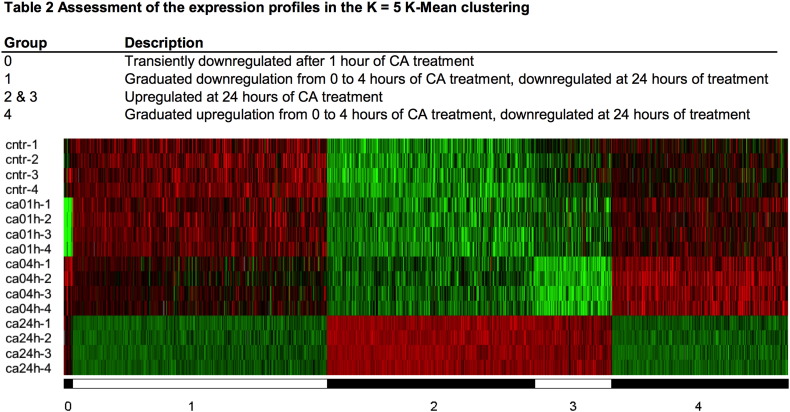
Heat map of the K-Mean clustering of differentially expressed transcripts. FDR < 10^3^ in ANOVA has been used as the criteria for differentially expression. Clustering has been performed with Euclidean distance measurement, K = 5 groups and 100 runs.

**Fig. 1:**
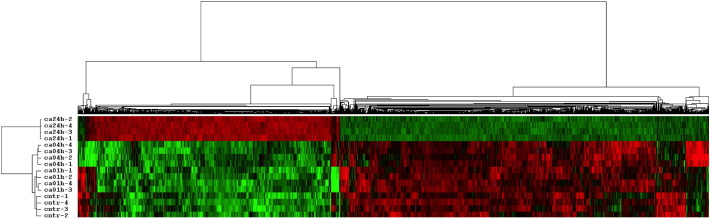
Hierarchical clustering of the differentially expressed probe-sets. Hierarchical clustering of the 1517 differentially expressed probe-sets identified the time dependent effect of calcium on Caco-2/AQ cells. Cntr refers to control; ca01h, ca04h and ca24h refer to calcium treatment for 1, 4 and 24 h, respectively.

We have performed hierarchical clustering of the probe sets to identify the time-dependent effect of the [Ca^2+^]_o_ treatment. The cluster analysis has been carried out using cluster version 2.11 [45] applying mean-centering and normalization of genes and arrays before average linkage clustering using uncentered correlation (Fig. 1). The hierarchical clustering of the differentially expressed genes confirmed that the most pronounced changes occurred after 24 h treatment with [Ca^2+^]_o_. According to the expression profile of the hierarchical cluster and to the approximation of the number of clusters using the David-Bouldin procedure we performed a K-Mean clustering (k = 5) using Cluster version 3.0, with Euclidean distance measurement as similarity matrix, and 100 runs (Suppl. Fig. 2).

Table 1, Table 2 show the top up-regulated and down-regulated genes after 1, 4, and 24 h treatment with [Ca^2+^]_o_.

#### 3.1.1 Enrichment analysis using DAVID^3^

The functional analysis of the dataset of 1571 differentially expressed probe-sets using DAVID revealed that the majority of the biological processes affected by [Ca^2+^]_o_ were linked to regulation of DNA replication (4.9%, *p* = 2.6 × 10^− 25^), G1/S transition of mitotic cell cycle (3.6%, *p* = 1.2 × 10^− 20^), cell division (6.1%, *p* = 1.5 × 10^− 16^), initiation of DNA replication (1.8%, *p* = 9.9 × 10^− 16^) (Table 3). The differentially expressed probe-sets were enriched in molecular functions such as protein, ATP, DNA, and RNA binding, amino acid transmembrane transporter activity, and DNA helicase activity. Additionally, the gene ontology (GO) cell component analysis revealed that the differentially expressed probe-sets were significantly enriched in the nucleoplasm (27.6%, *p* = 9.1 × 10^− 28^), cytosol (27%, *p* = 1.4 × 10^− 12^), nucleus (37.3%, *p* = 3 × 10^− 11^), extracellular exosome (21.4%, *p* = 2 × 10^− 9^), nucleolus (8.6%, *p* = 5.7 × 10^− 9^), condensed chromosome kinetochore (2%, *p* = 2.4 × 10^− 8^), and cytoplasm (35.1%, *p* = 6.4 × 10^− 8^). The analysis showed that the differentially expressed probe-sets were significantly enriched in 10 pathways, as defined by the Kyoto Encyclopedia of Genes and Genomes (KEGG). These pathways are involved in DNA replication (1.9%, *p* = 3.2 × 10^− 13^), cell cycle (3%, *p* = 2.4 × 10^− 9^), mismatch repair (1.1%, *p* = 3.4 × 10^− 7^), p53 signaling pathway (1.7%, *p* = 3.9 × 10^− 6^) (Table 3).

The transcripts upregulated after 24 h [Ca^2+^]_o_ treatment were enriched in processes linked to nucleosome assembly, amino acid transport, and redox processes. The most upregulated gene in our study was the intestine-specific terminal differentiation marker sucrase-isomaltase, one of the typical differentiation markers of Caco-2 cells [46]. Other upregulated genes were genes involved in xenobiotic detoxification, ion transport, mechanisms linked to differentiation (Table 1).

Expression of genes involved in cell cycle regulation, purine and pyrimidine metabolism, RNA processing, translation, and protein degradation, were inhibited by [Ca^2+^]_o_ treatment. The majority of the genes that enriched these processes were downregulated after 24 h of [Ca^2+^]_o_ treatment. The most downregulated gene was transgelin, an actin-binding protein that increases metastatic potential of colon cancer cells [47], followed by asparagine synthetase and the transcription factor E2F7 (Table 2).

#### 3.1.2 Enrichment analysis using Ingenuity Pathway Analysis^4^

Based on the Ingenuity Pathway Analysis, among the top diseases and disorders associated with an enrichment of the differentially expressed probe-sets after 24 h treatment with [Ca^2+^]_o_ were cancer, injuries, gastrointestinal diseases, diseases of the reproductive system. The molecular and cellular functions enriched were cell cycle, cellular growth and proliferation, cell death and survival, DNA replication, recombination, and repair, and cellular development. Organismal survival, tissue, embryonic, and organismal development were enriched significantly in the differentially expressed probe-sets (Table 4).

#### 3.1.3 Regulation of DNA replication licensing by extracellular calcium

**Fig. 2:**
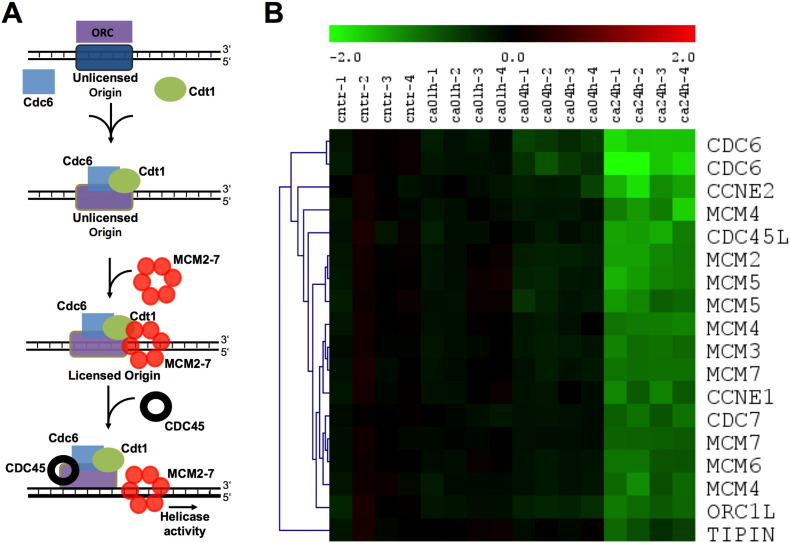
Regulation of DNA replication licensing by extracellular calcium. A: Schematic presentation of the replication licensing process. B: The expression of the DNA licensing factors is significantly inhibited by 24 hour treatment with 2 mM [Ca^2+^]_o_ (ca24h). Four replicates were analyzed for each time-point.

As most of the pathways and functions affected by the [Ca^2+^]_o_ treatment were linked to regulation of the cell cycle, we focused on the first steps in G1/S phase: the start of DNA replication, a process known as DNA replication licensing (Fig. 2A). Events of the replication licensing are precisely choreographed: first, origin recognition complex (ORC) binds to the DNA at the replication origins. DNA-bound ORC is required to recruit chromatin licensing and DNA replication factor 1 (CDT1) and cell division cycle 6 homolog (CDC6). This ORC-CDT1-CDC6 complex initiates the binding of the minichromosome maintenance complex (MCM) to the chromatin. Ten conserved proteins belong to the MCM group and are involved in DNA synthesis. MCM2–7 are related to each other, function as replicative helicases, and are involved in the initiation of eukaryotic genome replication [48]. Prior to the unwinding of the DNA, a series of protein complexes, such as cell division cycle 45 homolog (CDC45) are loaded to activate the helicases. CDC45 is pivotal for the transition to replication and it is required for the loading of several components of the DNA synthesis machinery including PCNA and DNA polymerase α and ε on to the chromatin [49].

The microarray analysis revealed that [Ca^2+^]_o_ treatment down-regulated expression of most of the genes involved in replication licensing (Fig. 2B). CDT1, although not among the top 1571 transcripts chosen for analysis, was gradually downregulated by [Ca^2+^]_o_ treatment, with an overall ANOVA *p* < 4.1 × 10^− 5^ with a maximum of 1.76 fold reduction after 24 h of [Ca^2+^]_o_ treatment.

### 3.2 Validation of the effect of extracellular calcium on replication licensing factors *in vitro*

**Fig. 3:**
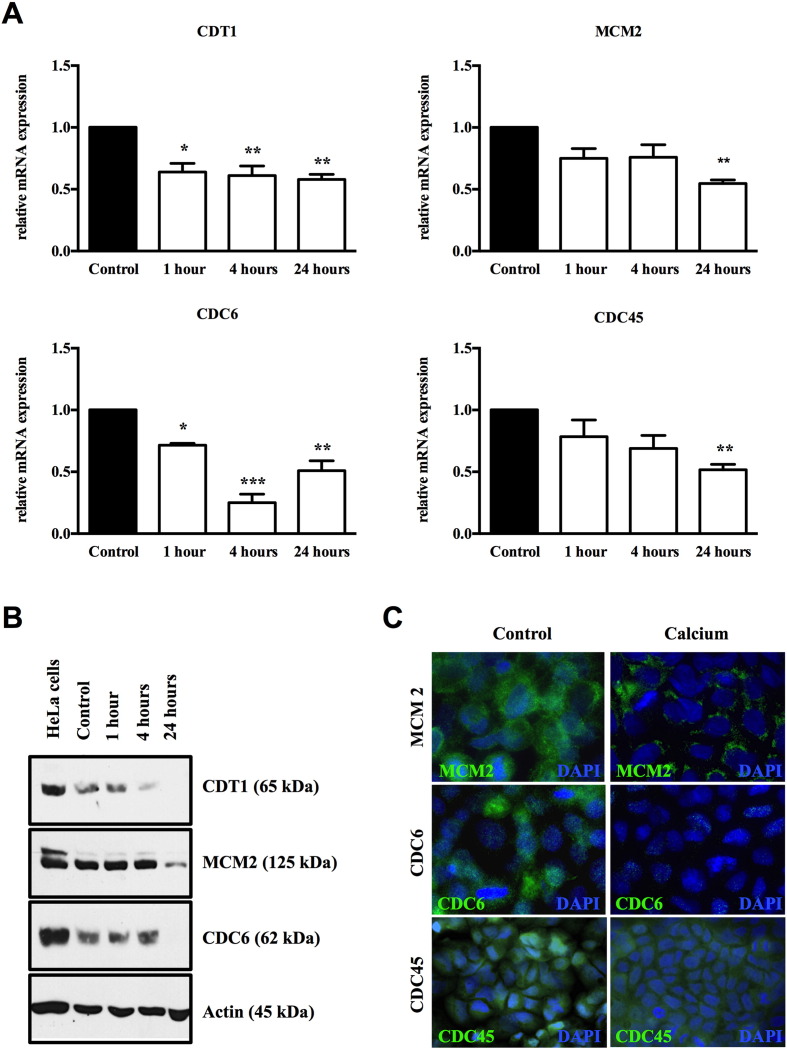
Confirmation of the microarray data in Caco-2/AQ cells. A: qRT-PCR analysis of the expression of CDT1, MCM2, CDC6, and CDC45. Bars represent mean ± SEM. Statistical significance was calculated using ANOVA followed by Tukey's post-test. Asterisks above bars indicate significant changes within groups, *p < 0.05, **p < 0.01, ***p < 0.001. B: Representative Western Blot after treatment of the cells for 24 h with 2 mM [Ca^2+^]_o_. C: Representative immunofluorescent images of MCM2, CDC6, and CDC45 (green) after treatment of Caco-2/AQ cells with 2 mM [Ca^2+^]_o_ for 24 h. Nuclei are stained with DAPI.

We validated the inhibition of DNA licensing *in vitro* at both mRNA and protein level. Treatment of Caco-2/AQ cells with 2 mM [Ca^2+^]_o_ for 1, 4 and 24 h significantly downregulated mRNA expression of key components of the DNA replication licensing signature, CDT1, CDC6, CDC45, and MCM2. (Fig. 3A). We further examined whether the effects observed were also translated at protein level. 24 h treatment with [Ca^2+^]_o_ inhibited CDT1, CDC6, CDC45, and MCM2 protein expression, as measured by immunofluorescence and western blot (Fig. 3B and C).

### 3.3 Effect of dietary calcium on replication licensing *in vivo*

**Fig. 4:**
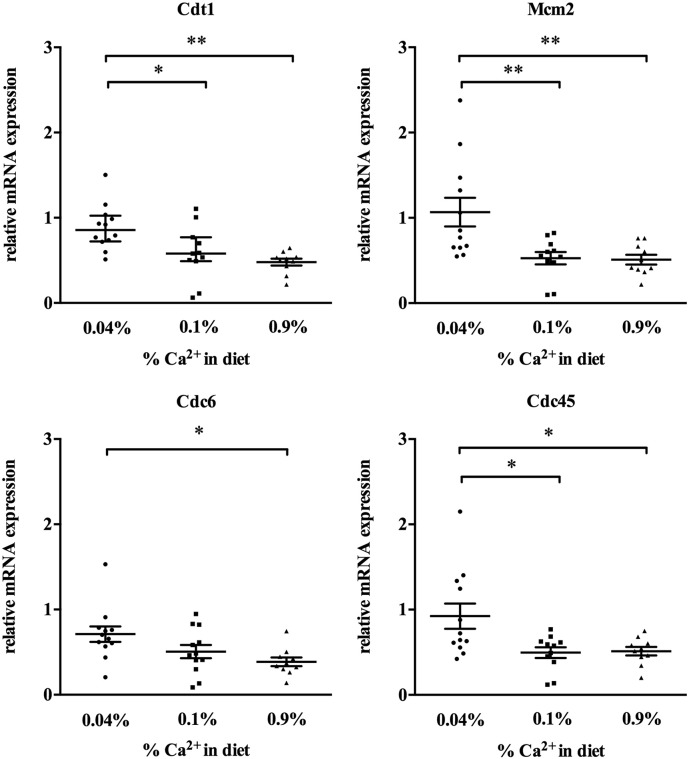
Effect of dietary calcium on the expression of licensing factors in the colon of mice. Effect of dietary calcium on the expression of licensing factors in the colon of mice (*n* = 10 mice/group) fed for 8 months with AIN76 diet containing 0.04%, 0.1%, or 0.9% calcium. Individual data points are shown ± SEM. Statistical significance was calculated using ANOVA followed by Tukey's post-test. Asterisks indicate significant changes within groups, *p < 0.05, **p < 0.01.

In order to test whether dietary calcium inhibits DNA replication also *in vivo* in the colon of mice, we assessed the expression of the genes regulating replication licensing (CDT1, CDC6, CDC45, and MCM2) in the colon of C57BL/6 mice fed with a diet containing high (0.9%), standard (0.1%) or low (0.04%) levels of dietary calcium for 8 months (at least 10 mice/group). Expression levels of all genes examined were significantly higher in the colon of mice fed 0.04% calcium whereas the lowest expression levels were found in the colon of mice fed with 0.9% calcium (Fig. 4).

### 3.4 The calcium-sensing receptor (CaSR) suppresses replication licensing in colon cancer cells

**Fig. 5:**
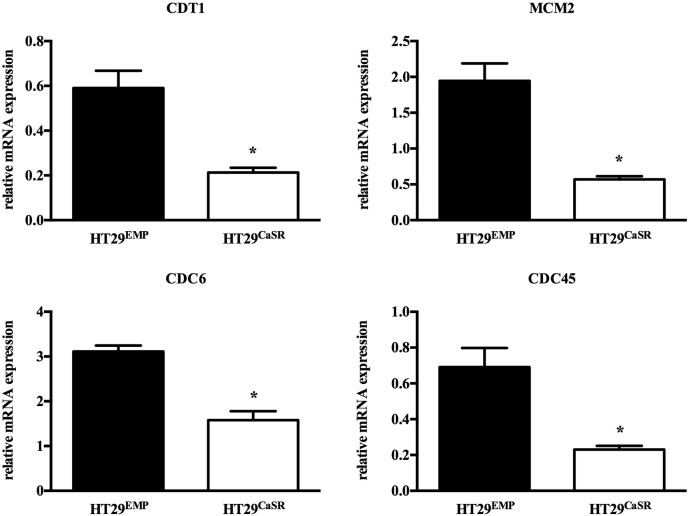
Expression of licensing factors in HT29 colon cancer cells overexpressing CaSR. Overexpression of CaSR in HT29 colon cancer cells (HT29^CaSR^) significantly inhibited the expression of CDT1, MCM2, CDC6, and CDC45 compared with the controls transfected with an empty vector (HT29^Emp^). Bars represent mean ± SEM. Statistical significance was calculated using Student's *t*-test. Asterisks above bars indicate significant changes compared with control cells, *p < 0.05.

We have shown previously that the anti-proliferative, pro-differentiating effect of [Ca^2+^]_o_ in colonocytes is mediated by the CaSR [16], [31], [32], [34]. In order to test whether the CaSR mediates also the effect of [Ca^2+^]_o_ on replication licensing we used the HT29 cell line stably transfected either with the full length CaSR (HT29^CaSR^) or with an empty vector (HT29^Emp^). In contrast to Caco-2 cells, parental HT29 cells are not responsive to the antiproliferative effect of [Ca^2+^]_o_ [33] and express undetectable levels of CaSR [34]. The HT29^CaSR^ cells overexpressing the CaSR, expressed significantly lower levels of CDT1, CDC6, CDC45, and MCM2 compared with the HT29^Emp^ controls (Fig. 5). Moreover, we have shown recently, that the expression of the licensing factors is significantly lower in the colon of mice expressing the CaSR when compared with mice lacking CaSR systemically [34].

## 4 Discussion

We examined the effects of [Ca^2+^]_o_ *in vitro* and *in vivo* to understand the protective effects of calcium in colorectal tumorigenesis and to determine molecular pathways that may be affected. We focused on the effects of calcium on replication licensing as replicative stress caused by mistakes during DNA replication is one of the crucial, early characteristics of neoplastic transformation. Our studies revealed that [Ca^2+^]_o_ regulated critical steps of the replication licensing both *in vitro* and *in vivo* preventing incorrect firing of replication, and that the CaSR is needed for an errorless licensing process.

Calcium signaling integrates with other signal-transduction cascades to control a variety of processes, including transcriptional regulation and cellular proliferation [50]. Calcium is universally required throughout the mammalian cell cycle and is especially important early in G1, at the G1/S and G2/M transitions [50], and the requirement for [Ca^2+^]_o_ is cell-type dependent. Colonocytes need very low levels of [Ca^2+^]_o_ to proliferate optimally and they turn off their proliferation in the presence of extracellular calcium in the range of 0.8–2.2 mM [20], [51]. The molecular mechanisms governing calcium-dependent pathways regulating cell growth and differentiation are still poorly understood [52]. It seems that the calcium-dependence of colorectal tumor cells changes. On one hand, those Ca^2+^-dependent processes that inhibit proliferation are suppressed in colorectal tumors [53]. On the other hand, Ca^2+^-dependent processes that correlate with increased cell proliferation, invasion, and survival of tumor cells, such as store-operated Ca^2+^ entry and store-operated currents are largely enhanced in tumor cells, while the level of Ca^2+^ in internal stores is low [54].

In order to gain an insight into the complex, stepwise alterations in gene expression after [Ca^2+^]_o_ treatment, we performed microarray gene expression profiling. The Caco-2/AQ cells, used as a model system in the microarray study, are able to differentiate spontaneously in culture, gaining an enterocyte-like phenotype. The molecular changes that occur during this differentiation process have been characterized extensively [29], [55], [56], [57]. The microarray analysis of the 54,675 probe-sets assessed in the present study revealed that treatment of Caco-2/AQ cells with 2 mM [Ca^2+^]_o_ led to changes in gene expression pattern very similar to the changes observed during the spontaneous differentiation of these cells [29], [30]. These changes were similar also to those observed during the differentiation of colonocytes while migrating from the bottom towards the top of the colonic crypt [29], [30]. This suggests that [Ca^2+^]_o_ treatment supports the natural differentiation process of colonocytes. Interestingly, numerous genes inhibited by [Ca^2+^]_o_ were found to be upregulated both in the embryonal development of the colon and in colorectal tumorigenesis [58].

The unbiased approach of gene expression profiling revealed that [Ca^2+^]_o_ significantly inhibited processes and pathways linked to DNA replication. Replicative stress, caused by mistakes in DNA replication, will lead to genetic instability, one of the drivers of colorectal tumorigenesis [59]. Replication of DNA is intricately regulated in all cell types, beginning with the licensing at the sites of origin of replication. The events during replication licensing, periodic assembly and disassembly of the pre-replication complex at replication origins, ensure that replication takes part once and only once during one cell cycle (Fig. 2A, [60], [61], [62], [63]).

Extracellular calcium treatment suppressed replication licensing and all components were significantly downregulated, implying a controlled inhibition of replication. Upregulation of the expression level of the MCM2–7 complex is an early event in multi-step tumorigenesis. It was found in several premalignant and malignant lesions, and has been linked to reduced survival in several tumor types (for rev. see *e.g.* [64]). Genes encoding MCM proteins are present in poor prognostic signatures for a range of malignancies [65], [66]. High levels of all six MCM proteins were strongly associated with shorter survival of breast cancer patients [67]. Overexpression of the MCMs significantly correlated with pancreatic cancer progression and prognosis [68], cervical carcinogenesis [69], and poor outcome of glioma patients [70]. In colorectal tumors MCM2 expression significantly associated with histological grade, Dukes' stage, and existence of metastases [71], therefore it could become an indicator for the management of colorectal cancer patients. High levels of the helicase MCM7 and the polymerase theta POLQ in colorectal tumors were significantly linked to poor patient survival [66]. We found that [Ca^2+^]_o_ treatment reduced the expression of these genes in parallel with the expression of other licensing factors. Interestingly, MCM9, which has a role in the S-phase checkpoint pathways and is often suppressed in colorectal tumors, was upregulated by [Ca^2+^]_o_ treatment. The physiological relevance of our findings is mirrored in the *in vivo* experiment where low dietary calcium led to increased levels of the licensing factors in the colon of mice, while high calcium intake was able to prevent this upregulation. We have shown previously that even shorter exposure to low calcium diet increases colonic proliferation [17], [26].

It is known that epithelial cells decrease their proliferation when they form cell-cell contacts. Therefore, the obvious explanation for the anti-proliferative effect of [Ca^2+^]_o_ would be that of contact inhibition upon restoration of cell-cell contacts. However, we have shown previously [72] that proliferation of Caco-2 cells was inhibited also by gadolinium, an agonist of the calcium-sensing receptor, that has no influence on cell-cell contact, suggesting a role for the CaSR. Indeed, restoration of CaSR expression or function inhibited proliferation of colorectal cancer cells [34]. The involvement of the CaSR in mediating the anti-proliferative effects of [Ca^2+^]_o_ in the colon was also described by our previous observation of a significant inverse correlation between the expression of the CaSR and the expression of replication licensing factors in colorectal tumors [34]. To address whether the CaSR, a tumor suppressor in the colon, was involved in regulating the DNA replication machinery, we transfected the HT29 colon cancer cells, which have very low endogenous CaSR levels, with the full-length CaSR. Indeed, expression levels of the factors involved in DNA replication licensing were significantly lower in cells overexpressing the CaSR. Furthermore, we have recently reported that the colon of mice lacking the CaSR have significantly higher expression levels of the genes that control replication licensing compared with the controls [34].

Genetic instability is a main driver of colorectal cancer. As preservation of genome integrity will limit cancer risk, DNA replication is becoming an important factor in cancer. Most genes involved in replication licensing are aberrantly expressed in colorectal tumors compared with the normal tissue [66]. Repression of replication licensing seems to be the ubiquitous route by which the proliferative capacity of cells is lowered. Our study suggests that one of the cancer preventive mechanisms of [Ca^2+^]_o_ is maintenance of faithful DNA duplication, underlining the importance of adequate dietary calcium intake. Calcium is an inexpensive and easily available substance that prevents activation of replication in the colon and suppresses expression of genes that would lead to development of aggressive tumors. As the CaSR mediates most of the tumor preventive effects of [Ca^2+^]_o_, the level of the CaSR could become a marker for the responsiveness to the anti-proliferative effect of [Ca^2+^]_o_.

The following are the supplementary data related to this article.

Suppl. Table 1 Gene expression analysis of the 54,675 probe sets. Suppl. Table 1

## Transparency Document

Transparency document. Image 1

## Figures and Tables

**Table 1:** List of the top genes up-regulated by [Ca^2+^]_o_ treatment: Top 10 for each time point. *p* value assed based on pair-wise comparisons with the untreated controls using Student's *t*-test (two sided, unpaired, assuming unequal variances).

Name	Symbol	Fold change	*p*-Value
*Increased after 1* *h*			
Chemokine (C-X-C motif) ligand 2	CXCL2	1.335	0.0002
Jun oncogene	JUN	1.329	0.0080
Chemokine (C-X-C motif) ligand 3	CXCL3	1.323	0.0062
RAB27B, member RAS oncogene family	RAB27B	1.290	0.0057
Nuclear receptor coactivator 7	NCOA7	1.233	0.0064
EPH receptor A2	EPHA2	1.223	0.0264

*Increased after 4* *h*			
Inhibitor of DNA binding 1, dominant negative helix-loop-helix protein	ID1	1.398	0.0374
Growth arrest-specific 5	GAS5	1.388	0.0015
Cadherin 7, type 2	CDH7	1.348	0.0095
v-myc myelocytomatosis viral oncogene homolog (avian)	MYC	1.266	0.0103
WD repeat domain 43	WDR43	1.263	0.0012
Cyclin Y-like 1	CCNYL1	1.260	0.0123
Partner of NOB1 homolog (*S. cerevisiae*)	PNO1	1.241	0.0029
BRCA2 and CDKN1A interacting protein	BCCIP	1.241	0.0083
SLIT-ROBO Rho GTPase activating protein 1	SRGAP1	1.238	0.0006
Phospholipase D1, phosphatidylcholine-specific	PLD1	1.230	0.0000

*Increased after 24* *h*			
Sucrase-isomaltase (alpha-glucosidase)	SI	5.320	1.17E−05
RAB27B, member RAS oncogene family	RAB27B	3.581	7.43E−03
Klotho beta	KLB	3.297	1.95E−03
Regenerating islet-derived family, member 4	REG4	3.211	6.46E−04
Monoamine oxidase A	MAOA	3.001	2.03E−03
Potassium inwardly-rectifying channel, subfamily J, member 16	KCNJ16	2.641	5.11E−06
Aldo-keto reductase family 1, member D1	AKR1D1	2.617	1.31E−04
Similar to calpain 8	LOC388743	2.479	1.63E−03
Fibrinogen beta chain	FGB	2.412	1.12E−03
Lectin, galactoside-binding, soluble, 7 (galectin 7)	LGALS7	2.366	6.14E−03

**Table 2:** List of the top genes down-regulated by [Ca^2+^]_o_ treatment: Top 10 for each time point. *p* value assed based on pair-wise comparisons with the untreated controls using Student's *t*-test (two sided, unpaired, assuming unequal variances).

Name	Symbol	Fold change	*p*-Value
*Decreased after 1 h*			
DNA-damage-inducible transcript 4	DDIT4	−2.265	0.0048
Ring finger protein 186	RNF186	−1.807	0.0000
Stanniocalcin 2	STC2	−1.531	0.0022
Protein phosphatase 2regulatory subunit B, alpha isoform	PPP2R2A	−1.505	0.0069
Hematopoietically expressed homeobox	HHEX	−1.455	0.0086
Forkhead box P1	FOXP1	−1.427	0.0016
Stanniocalcin 1	STC1	−1.343	0.0013
Polymerase (DNA directed), theta	POLQ	−1.333	0.0044
Solute carrier family 30 (zinc transporter), member 1	SLC30A1	−1.319	0.0046
Kinesin family member 15	KIF15	−1.313	0.0148

*Decreased after 4* *h*			
Metallothionein 1X	MT1X	−1.720	0.00754
Metallothionein 1F	MT1F	−1.713	0.00429
Jun oncogene	JUN	−1.688	0.00075
Activating transcription factor 3	ATF3	−1.675	0.00011
Meiotic nuclear divisions 1 homolog (*S. cerevisiae*)	MND1	−1.554	0.00082
Tensin 1	TNS1	−1.544	0.00021
Denticleless homolog (*Drosophila*)	DTL	−1.504	0.00740
Cell division cycle 6 homolog (*S. cerevisiae*)	CDC6	−1.497	0.00285
Achaete-scute complex homolog 2 (*Drosophila*)	ASCL2	−1.488	0.00033

*Decreased after 24* *h*			
Transgelin	TAGLN	−7.575	7.10E−06
Asparagine synthetase	ASNS	−4.283	9.94E−07
E2F transcription factor 7	E2F7	−3.947	1.82E−03
Denticleless homolog (*Drosophila*)	DTL	−3.653	3.61E−05
Solute carrier family 7, (cationic amino acid transporter, y + system) member 11	SLC7A11	−3.580	8.37E−04
Metallothionein 1F	MT1F	−3.552	3.62E−05
Metallothionein 1X	MT1X	−3.414	9.34E−06
Minichromosome maintenance complex component 10	MCM10	−3.349	6.85E−05
Phosphoserine aminotransferase 1	PSAT1	−3.309	4.66E−05
Cell division cycle 6 homolog (*S. cerevisiae*)	CDC6	−3.090	7.49E−06

**Table 3:** Gene ontology terms and pathways enriched by the differentially expressed probe-sets using DAVID.

Category	Term	Gene count	%	*p*-Value	FDR q value
*Biological processes*					
GOTERM_BP_DIRECT	DNA replication	54	4.9	2.6E−25	9.2E−22
GOTERM_BP_DIRECT	G1/S transition of mitotic cell cycle	39	3.6	1.2E−20	2.1E−17
GOTERM_BP_DIRECT	Cell division	67	6.1	1.5E−16	1.3E−13
GOTERM_BP_DIRECT	DNA replication initiation	20	1.8	9.9E−16	8.7E−13
GOTERM_BP_DIRECT	Mitotic nuclear division	53	4.8	5.8E−15	4.1E−12
GOTERM_BP_DIRECT	Sister chromatid cohesion	28	2.6	7.3E−11	4.3E−8
GOTERM_BP_DIRECT	Telomere maintenance *via* recombination	16	1.5	2.0E−10	1.0E−7
GOTERM_BP_DIRECT	Cell proliferation	54	4.9	4.8E−9	2.1E−6
GOTERM_BP_DIRECT	Amino acid transport	14	1.3	4.4E−8	1.7E−5
GOTERM_BP_DIRECT	DNA strand elongation involved in DNA replication	10	0.9	5.7E−8	2.0E−5
GOTERM_BP_DIRECT	G2M transition of mitotic cell cycle	28	2.6	6.0E−8	1.9E−5
GOTERM_BP_DIRECT	tRNA aminoacylation for protein translation	14	1.3	3.1E−7	9.0E−5
GOTERM_BP_DIRECT	Regulation of transcription involved in G1/S transition of mitotic cell cycle	11	1.0	3.3E−7	8.8E−5

*Molecular functions*					
GOTERM_MF_DIRECT	Protein binding	506	46.1	1.5E−15	1.6E−12
GOTERM_MF_DIRECT	Poly(A) RNA binding	118	10.7	1.6E−8	9.1E−6
GOTERM_MF_DIRECT	Amino acid transmembrane transporter activity	15	1.4	3.0E−7	1.1E−4
GOTERM_MF_DIRECT	Single-stranded DNA-dependent ATPase activity	8	0.7	4.9E−7	1.4E−4
GOTERM_MF_DIRECT	Identical protein binding	70	6.4	1.3E−6	2.8E−4
GOTERM_MF_DIRECT	ATP binding	136	12.4	3.4E−6	6.5E−4
GOTERM_MF_DIRECT	Damaged DNA binding	16	1.5	4.8E−6	7.8E−4
GOTERM_MF_DIRECT	DNA replication origin binding	7	0.6	2.6E−5	3.7E−3
GOTERM_MF_DIRECT	Single-stranded DNA binding	18	1.6	4.5E−5	5.7E−3
GOTERM_MF_DIRECT	DNA helicase activity	9	0.8	9.1E−5	1.0E−2

*GO cell component*					
GOTERM_CC_DIRECT	Nucleoplasm	303	27.6	9.1E−28	5.8E−25
GOTERM_CC_DIRECT	Cytosol	296	27.0	1.4E−12	4.6E−10
GOTERM_CC_DIRECT	Nucleus	410	37.3	3.0E−11	6.4E−9
GOTERM_CC_DIRECT	Extracellular exosome	235	21.4	2.0E−9	3.2E−7
GOTERM_CC_DIRECT	Nucleolus	94	8.6	5.7E−9	7.2E−7
GOTERM_CC_DIRECT	Condensed chromosome kinetochore	22	2.0	2.4E−8	2.5E−6
GOTERM_CC_DIRECT	Cytoplasm	385	35.1	6.4E−8	5.8E−6
GOTERM_CC_DIRECT	Nucleosome	18	1.6	1.2E−6	9.4E−5
GOTERM_CC_DIRECT	Nuclear chromosome, telomeric region	23	2.1	3.2E−6	2.3E−4
GOTERM_CC_DIRECT	Chromatin	19	1.7	1.5E−5	9.2E−4
GOTERM_CC_DIRECT	Kinetochore	17	1.5	2.0E−5	1.2E−3
GOTERM_CC_DIRECT	MCM complex	6	0.5	4.4E−5	2.3E−3

*Pathways*					
KEGG_PATHWAY	DNA replication	21	1.9	3.2E−13	8.6E−11
KEGG_PATHWAY	Cell cycle	33	3.0	2.4E−9	3.1E−7
KEGG_PATHWAY	Mismatch repair	12	1.1	3.4E−7	3.0E−5
KEGG_PATHWAY	P53 signaling pathway	19	1.7	3.9E−6	2.6E−4
KEGG_PATHWAY	Nucleotide excision repair	14	1.3	3.4E−5	1.8E−3
KEGG_PATHWAY	Biosynthesis of antibiotics	35	3.2	5.7E−5	2.5E−3
KEGG_PATHWAY	Fanconi anemia pathway	14	1.3	1.2E−4	4.5E−3
KEGG_PATHWAY	Aminoacyl-tRNA biosynthesis	12	1.1	3.5E−4	1.2E−2
KEGG_PATHWAY	Carbon metabolism	20	1.8	1.7E−3	4.8E−2
KEGG_PATHWAY	Proteasome	11	1.0	1.8E−3	4.6E−2

**Table 4:** Top biological functions and canonical pathways in Ingenuity (www.ingenuity.com) affected after 24 h treatment with 2 mM [Ca^2+^]_o_.

Category	Term	*p*-Value	No. of molecules
Top biological functions: Diseases and disorders			
	Cancer	2.25E−04−3.32E−19	720
	Organismal injury and abnormalities	2.25E−04−3.32E−19	723
	Reproductive system disease	1.99E−04−2.21E−16	422
	Gastrointestinal disease	1.99E−04−5.05E−14	634
	Endocrine system disorders	1.54E−04−9.90E−11	158
Molecular and cellular functions			
	Cell cycle	2.18E−04−1.05E−28	224
	Cellular growth and proliferation	2.30E−04−6.58E−26	336
	Cell death and survival	2.21E−04−3.03E−24	334
	DNA replication, recombination, and repair	2.35E−04−6.28E−20	183
	Cellular development	2.18E−04−6.02E−14	272
Physiological system development and function			
	Organismal survival	1.94E−05−4.02E−16	234
	Connective tissue development and function	2.18E−04−1.74E−09	128
	Tissue development	2.30E−04−1.74E−09	141
	Embryonic development	1.99E−04−8.84E−08	161
	Organismal development	1.99E−04−4.25E−07	199
Top canonical pathways:			Overlap
	Cell cycle control of chromosomal replication	1.01E−17	50%
	Role of BRCA1 in DNA damage response	5.22E−14	28.2%
	Estrogen-mediated S-phase Entry	1.15E−11	50%
	Mismatch repair in eukaryotes	3.12E−11	62.5%
	Hereditary breast cancer signaling	8.84E−11	17.6%

## Footnotes

3 DAVID version 6.8 (20.10.2016).

4 Analysis on 20.10.2016.
